# Dual Immune Lineage Activation Underlies Systemic Immunopathology in Acute-on-Chronic Liver Failure

**DOI:** 10.1016/j.ajpath.2026.03.012

**Published:** 2026-04-18

**Authors:** Leah Spade, Aroma Chanda, Kamal Baral, Ramsey Rohner, Aidan J. Thomas, Bilon Khambu

**Affiliations:** Department of Pathology and Laboratory Medicine, Tulane University School of Medicine, New Orleans, Louisiana

## Abstract

Acute-on-chronic liver failure (ACLF) is a rapidly progressive syndrome characterized by acute hepatic decompensation in the setting of chronic liver disease and frequently accompanied by multi-organ dysfunction/failure and high short-term mortality. Although a dysregulated immunity (immunopathology) is increasingly recognized as a central driver of ACLF pathogenesis, the mechanistic basis linking immune activation to systemic organ injury remains poorly defined, in part due to the lack of a preclinical model that faithfully recapitulates immune-mediated organ damage. Here, a combined carbon tetrachloride/acetaminophen/lipopolysaccharide model (CALPS) was used that integrates chronic hepatotoxic injury with acute metabolic (acetaminophen) and inflammatory (lipopolysaccharide) stressors to reproduce key clinical and immunologic features of ACLF. CALPS mice developed severe hepatic injury, advanced fibrosis, and marked systemic inflammation. Comprehensive immunophenotyping revealed concurrent activation of myeloid and lymphoid lineages, accompanied by extensive infiltration of neutrophils, monocytes/macrophages, and T lymphocytes into the liver and extrahepatic organs, including kidney, lung, and heart. These immune abnormalities were specific to ACLF and absent in chronic liver disease, acute liver failure, and control cohorts. Notably, renal dysfunction correlated strongly with immune cell infiltration, supporting a mechanistic link between pathologic immune activation and multi-organ failure. Collectively, the CALPS model provides a robust and translational platform to investigate immune-mediated organ injury in ACLF and to advance targeted immunomodulatory therapies.

## Graphical abstract

**Figure:**
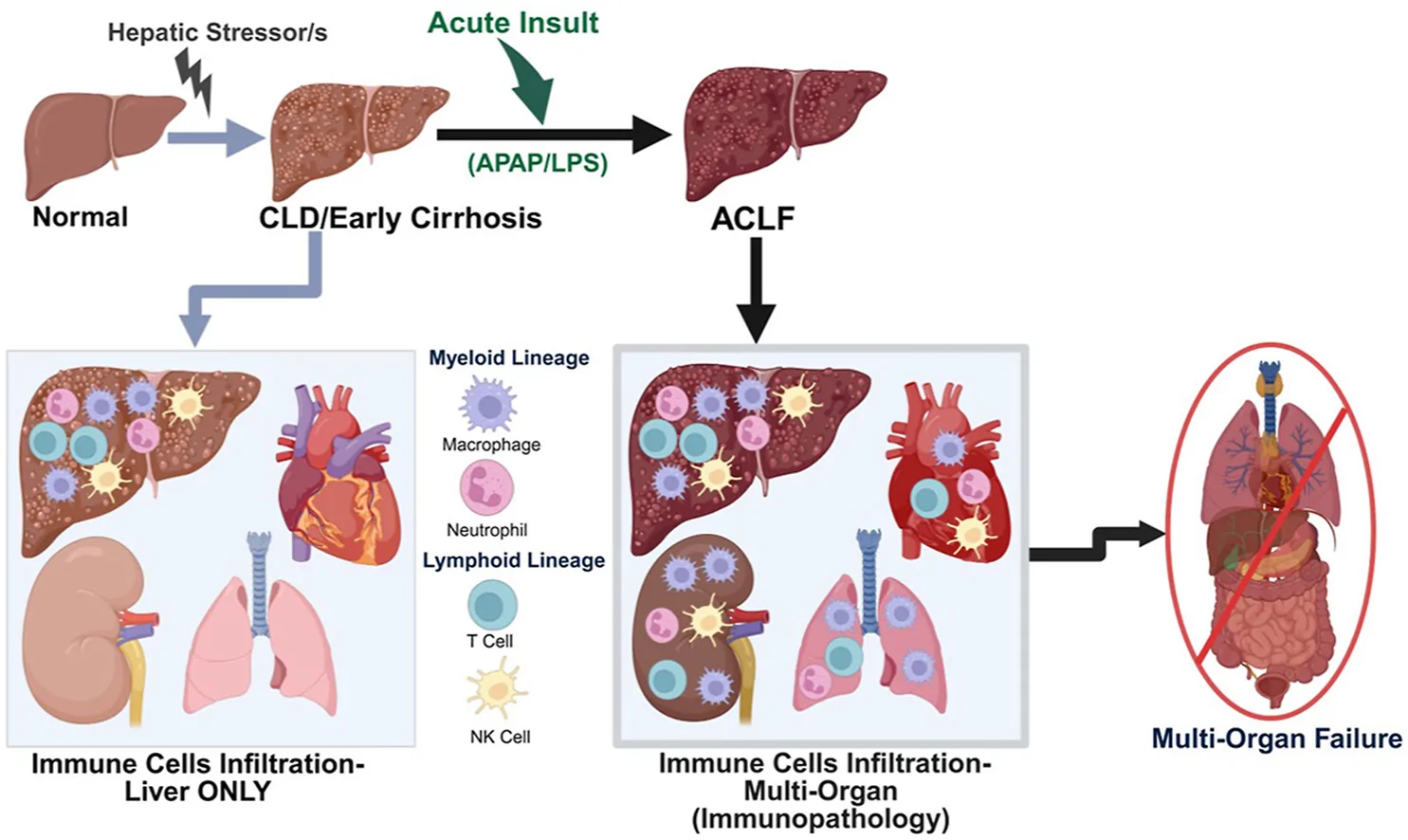

Acute-on-chronic liver failure (ACLF) is a rapidly progressive and life-threatening clinical syndrome characterized by acute hepatic decompensation in patients with underlying chronic liver disease (CLD), with or without cirrhosis.^1^, ^2^, ^3^, ^4^, ^5^ ACLF often culminates in multi-organ dysfunction or multi-organ failure (MOF) and carries a high short-term mortality rate. Despite its clinical importance, the underlying pathophysiological mechanisms remain incompletely understood.

Emerging evidence indicates that immunopathology, defined as immune-mediated tissue damage caused by inappropriate activation of immune pathways, plays a central role in ACLF and its systemic complications. Immunopathology leads to structural and functional organ alterations, accelerating tissue injury and creating a vicious cycle that ultimately results in organ dysfunction and failure.^6^ Systemic hyperinflammation, a hallmark of ACLF, reflects this immunopathologic process.^7^^,^^8^ Although clinical studies suggest that immunopathology contributes to MOF and mortality in ACLF,^6^^,^^9^, ^10^, ^11^, ^12^ direct experimental evidence establishing causality is limited, and the precise mechanisms remain poorly defined. Understanding these pathways is essential for developing targeted interventions to restore immune homeostasis and mitigate organ injury. A major barrier to progress is the lack of a well-characterized preclinical model that faithfully reproduces the immunopathologic and clinical features of ACLF.

Preclinical models are indispensable for dissecting disease mechanisms because they allow controlled investigation of dynamic immune changes during ACLF progression, including cytokine storms and immune cell activation.^13^^,^^14^ Animal models enable temporal tissue sampling and detailed immunophenotyping, which are challenging in human studies due to ethical and logistical constraints. To address this gap, the carbon tetrachloride/acetaminophen/lipopolysaccharide [CCl_4_/APAP/LPS (CALPS)] model was used, which combines chronic liver injury induced by CCl_4_ with an acute hepatotoxic insult (APAP) and a systemic inflammatory trigger (LPS).^15^ This “acute-on-chronic” multi-hit approach closely mimics ACLF pathophysiology, producing severe hepatic necrosis, impaired regeneration, and coagulopathy.

The CALPS model has emerged as one of the most clinically relevant experimental systems for studying ACLF.^13^ In this model, chronic liver injury is induced by repeated administration of CCl_4_, followed by an acute hepatotoxic insult with APAP and a systemic inflammatory trigger using LPS. This “acute-on-chronic” double-hit approach closely recapitulates ACLF pathophysiology, resulting in severe hepatic necrosis, impaired regeneration, and coagulopathy. CALPS animals exhibit hallmark clinical features, including hyperbilirubinemia, prolonged prothrombin time, ascites, portal hypertension, jaundice, coagulopathy, and elevated blood ammonia levels.^15^

The current study includes a comprehensive immunopathologic assessment of the CALPS model. Our findings show extensive immune cell infiltration not only in the liver but also in extrahepatic organs such as the lung, kidney, and heart. Both myeloid lineage cells (neutrophils and monocytes/macrophages) and lymphoid lineage cells (T lymphocytes) were markedly recruited to these tissues. Importantly, this widespread immune infiltration was observed exclusively in ACLF animals and was absent in CLD, acute liver failure (ALF), or healthy controls. Furthermore, renal dysfunction strongly correlated with immune infiltration, highlighting a mechanistic link between immunopathology and MOF. These results establish the CALPS model as a clinically relevant platform for mechanistic studies and for developing targeted immunomodulatory strategies to mitigate immune-mediated injury in ACLF.

## Materials and Methods

### Animal Models

Wild-type C57BL/6 male mice (8 to 10 weeks old) were purchased from The Jackson Laboratory (Bar Harbor, ME). Atg7^F/F^ (RBRC02759) mice were obtained from the RIKEN BioResource Research Center (Ibaraki, Japan).^16^^,^^17^ To generate hepatocyte-specific Atg7-deficient (hepAtg7^−/−^) mice, Atg7^F/F^ mice were crossed with Alb-Cre mice.^18^, ^19^, ^20^, ^21^, ^22^ Mice were housed under a 12-hour light/dark cycle with *ad libitum* access to food and water. All animal procedures were approved by the Institutional Animal Care and Use Committee at Tulane University (Approval No. 1809). Ages of mice are specified in figure legends.

In this study, only male mice were used to reduce the biological variability associated with hormonal cycling in female mice, which can influence immune responses and metabolic injury pathways. This is acknowledged as a limitation, and future studies will include female mice to assess potential sex-specific differences in ACLF pathophysiology.

### Murine ACLF Model (CALPS)

**Figure 1:**
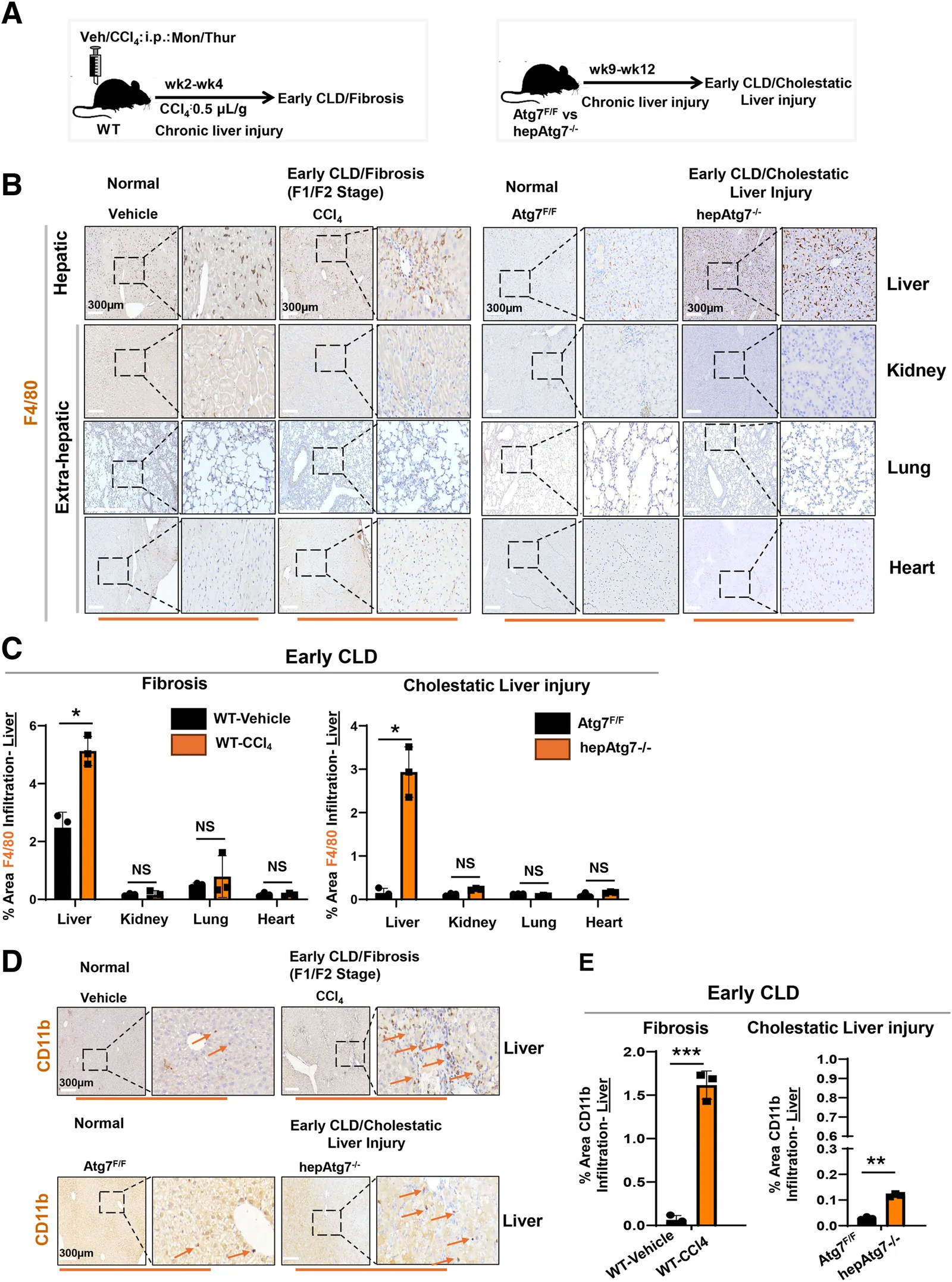
Early-stage chronic liver disease (CLD) does not exhibit extrahepatic immune infiltration. **A:** Experimental design for early CLD models: i) carbon tetrachloride (CCl_4_)-based model with chronic CCl_4_ administration (0.5 μL/g, i.p.) for 2 to 4 weeks; and ii) genetic model with hepatocyte-specific Atg7 deletion causing cholestatic liver injury. **B:** Immunohistochemical staining for F4/80–positive macrophages in liver, kidney, lung, and heart sections from both models, showing localized hepatic infiltration and absence of extrahepatic immune infiltration. **C:** Quantification of F4/80–positive area in liver versus extrahepatic tissues across early CLD models. **D:** Immunohistochemical staining for CD11b-positive myeloid cells in liver, kidney, lung, and heart sections from both models, confirming localized hepatic infiltration and absence of extrahepatic immune infiltration. **Arrows** indicate CD11b-positive cells. **E:** Quantification of CD11b-positive area in liver tissues across early CLD models. Bar graphs represent means ± SEM. *n* ≥ 5 per group. ∗*P* < 0.05, ∗∗*P* < 0.01, ∗∗∗*P* < 0.001. Scale bars = 300 μm (**B** and **D**). Original magnification, ×16 (**B** and **D**, **enlarged boxed areas**). NS, not significant; WT, wild type.

The CALPS model of ACLF was established as previously described.^15^ Briefly, chronic liver injury was induced by i.p. administration of CCl_4_ twice weekly at 0.1 µL/g for 5 weeks, followed by 0.5 µL/g for an additional 5 weeks. Acute liver injury was then triggered by a single dose of APAP (350 mg/kg, i.p.) combined with LPS (50 μg/kg, i.p.) to mimic an infectious insult (Figure 1A). This model recapitulates the clinical spectrum of ACLF, including progressive liver injury, inflammation, fibrosis, early cirrhosis, and hepatic decompensation (jaundice, coagulopathy, ascites, and hyperammonemia).^15^

### Early CLD Models

Early-stage CLD was modeled using: i) CCl_4_-based chronic injury—wild-type mice received CCl_4_ (0.5 μL/g, i.p.) for 2 to 4 weeks^23^; and ii) genetic cholestatic injury—hepatocyte-specific deletion of *Atg7*-induced cholestatic liver injury.^19^

### Tissue Collection and Processing

Liver, kidney, lung, and heart tissues were harvested and processed for paraffin embedding and cryosectioning. For paraffin blocks, tissues were fixed in 10% neutral-buffered formalin, dehydrated, cleared in xylene, and embedded in paraffin. For frozen blocks, tissues were fixed in 4% paraformaldehyde, cryoprotected in 30% sucrose, and embedded in Tissue-Plus O.C.T. compound (Thermo Fisher Scientific, Waltham, MA).

### Histology and Immunohistochemistry

Paraffin sections (4 μm) underwent hematoxylin and eosin staining and Sirius Red staining at the Tulane University Histology Research Laboratory. For immunohistochemistry, antigen retrieval was performed in citrate buffer (pH 6.0) at 95°C for 20 minutes. Sections were blocked with 10% goat serum and 1 M glycine in phosphate-buffered saline containing 0.5% Triton X-100 for 1 hour. Primary antibodies included CD11b (NB110-89474/dilution 1:100; Novus Biologicals, Littleton, CO), F4/80 (28463-1-AP/dilution 1:5000; Proteintech, Rosemont, IL), CD3 (557306/dilution 1:50; Pharmingen, San Diego, CA), CD56 (14255-1-AP/dilution 1:10,000; Proteintech), myeloperoxidase (MPO) (22225-1-AP/dilution 1:200; Proteintech), and lipocalin-2 (LCN2) (26991-1-AP/dilution 1:200; Proteintech). Slides were incubated overnight at 4°C, followed by incubation with horseradish peroxidase–conjugated secondary antibodies and developed using 3,3′-diaminobenzidine. Nuclei were counterstained with hematoxylin or Hoechst 33342.

### Image Acquisition and Quantification

Brightfield images were acquired by using an Olympus BX60 microscope equipped with a DP71 camera (Tokyo, Japan) or a Motic BA410 imaging system (Motic Instruments USA, Inc., Universal City, TX). All image quantification was performed in ImageJ2 version 1.x (NIH, Bethesda, MD; *http://imagej.net*) using identical exposure and acquisition settings across experimental groups.

For CD11b signal quantification in kidney tissue, quantitative image analysis with background subtraction was performed. Whole-slide images underwent color deconvolution (diaminobenzidine/hematoxylin), followed by application of a uniform intensity threshold that was predefined by using negative control sections. Diaminobenzidine-positive pixels were segmented and quantified as area fraction (percent diaminobenzidine-positive area per interstitial region of interest). Tubular lumina and glomerular tufts were excluded by using manually defined region of interest masks. All thresholds and regions of interest were applied consistently across all samples.

### General Histologic Analysis

Mouse liver tissues were fixed in 10% neutral-buffered formalin, embedded in paraffin, and sectioned. Hematoxylin and eosin staining and Sirius Red immunostaining were performed at the Tulane University Histology Research Laboratory.

Images were captured by using an Olympus BX60 microscope equipped with a DP71 color digital camera.

### Biochemical Assays

Serum alanine aminotransferase, aspartate aminotransferase, blood urea nitrogen, and creatinine levels were measured by using standard colorimetric kits (Thermo Fisher Scientific) according to manufacturer protocols.

### Statistical Analysis

Data are expressed as means ± SEM. Statistical significance was determined by using the two-tailed *t*-test for two-group comparisons. *P* < 0.05 was considered significant (∗*P* < 0.05, ∗∗*P* < 0.01, ∗∗∗*P* < 0.001).

## Results

### Early-Stage CLD Exhibits Localized Hepatic Inflammation without Extrahepatic Immune Infiltration

CLD is a progressive disorder that ultimately contributes to multiorgan dysfunction and failure, particularly involving the kidney, lung, and heart, and it represents a major cause of mortality. Clinically, CLD is classified as early-stage and advanced-stage disease. Although early-stage CLD is often associated with cardiovascular complications, advanced-stage CLD, frequently presented as ACLF, is characterized by systemic immunopathology and MOF, significantly increasing mortality risk. Understanding the transition from localized hepatic inflammation to systemic involvement is crucial because it reveals the mechanisms driving disease progression and identifies therapeutic targets to prevent multi-organ complications. Although early-stage hepatic inflammation in CLD is well established, whether extrahepatic inflammation also emerges during these initial phases remains unclear, a gap that could reshape early intervention strategies.

To address this gap, myeloid lineage immune cells in two distinct models of early-stage CLD were analyzed (Figure 1A): i) a CCl_4_-induced fibrosis model, in which wild-type mice received chronic doses of CCl_4_ (0.5 μL/g, i.p.) for 2 to 4 weeks; and ii) a genetic model of liver injury, in which hepatocyte-specific deletion of the *Atg7* gene resulted in cholestatic liver injury.^19^

Immunohistologic analysis revealed that both models displayed increased hepatic infiltration of F4/80–positive and CD11b-positive cells, consistent with localized liver inflammation (Figures 1, B-E). In contrast, these immune cell populations were not elevated in extrahepatic organs, including the kidney, lung, and heart, indicating the absence of systemic immune infiltration at this stage (Figures 1, B-E). Collectively, these findings indicate that early-stage CLD is defined by localized hepatic inflammation, with no evidence of widespread immunopathology. The transition from localized to systemic inflammation likely occurs as the disease progresses to an advanced stage such as ACLF.

### Exacerbated Liver Disease in ACLF Compared with CLD/Early Cirrhosis

To evaluate the progression from CLD to ACLF, hepatic and systemic pathologic changes in an advanced stage of CLD were examined. ACLF is a rapidly progressive and life-threatening syndrome characterized by acute hepatic decompensation in patients with underlying CLD, with or without cirrhosis, and is associated with multi-organ dysfunction and high short-term mortality.^1^, ^2^, ^3^, ^4^, ^5^ To model this condition, the CALPS model of ACLF, originally described by Nautiyal et al,^15^ was reproduced in this experimental system. Wild-type male mice (8 to 10 weeks old) were assigned to four groups: i) normal controls; ii) chronic liver disease (CLD/early cirrhosis) induced by 10 weeks of CCl_4_ administration; iii) ACLF induced by CCl_4_ followed by an acute insult with APAP and LPS; and iv) ALF induced by APAP plus LPS without prior chronic injury.

**Figure 2:**
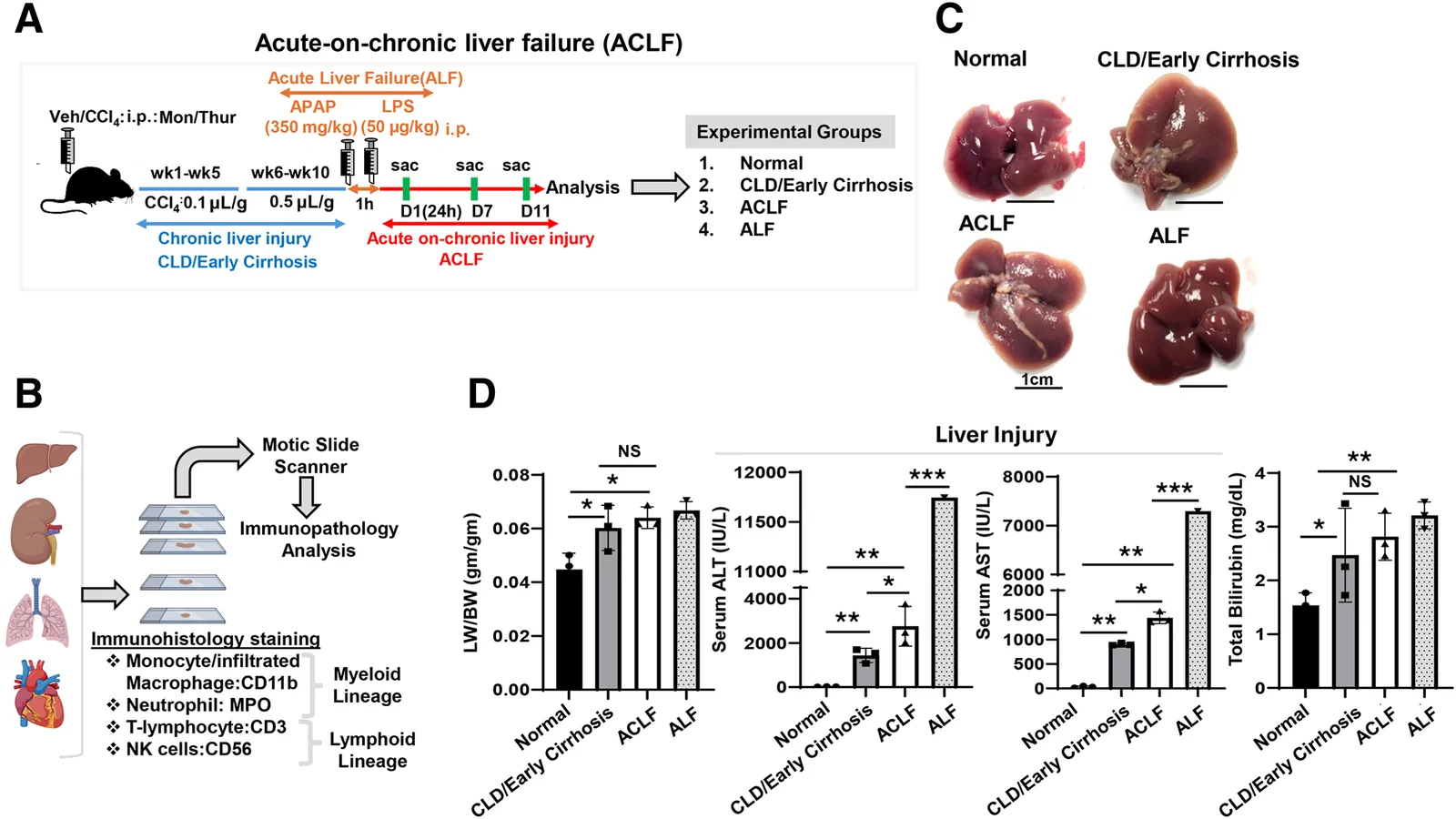
Exacerbated liver injury and associated pathologies in acute-on-chronic liver failure (ACLF). **A:** Schematic representation of the experimental design and time points for ACLF induction. Chronic liver disease (CLD)/early cirrhosis was established by i.p. administration of carbon tetrachloride (CCl_4_) for 10 weeks at a defined dose. Acute insult was induced by i.p. injection of acetaminophen (APAP; 350 mg/kg) and lipopolysaccharide (LPS; 50 μg/kg). Four experimental groups were analyzed: normal, CLD/early cirrhosis, ACLF, and acute liver failure (ALF). **B:** Overview of the experimental workflow for multi-organ histologic analysis. **C:** Representative gross liver images from each group. **D:** Liver weight-to-body weight ratio (LW/BW), serum liver injury markers [alanine aminotransferase (ALT), aspartate aminotransferase (AST), and total bilirubin levels] across groups. Data are expressed as means ± SEM. *n* ≥ 3 per group. ∗*P* < 0.05, ∗∗*P* < 0.01, ∗∗∗*P* < 0.001. Scale bars = 1 cm (**C**). NK, natural killer; NS, not significant.

Tissue samples were collected 24 hours after acute insult induction for histologic and immunopathologic analyses (Figure 2A). The focus was on the liver, kidney, lung, and heart (Figure 2B), as these organs represent clinically relevant targets of MOF in ACLF and are integral to disease grading systems.^2^^,^^5^ The liver was included because it is the primary site of injury and the initiating organ in ACLF, essential for validating hepatic pathology. The kidney was examined because renal dysfunction is a major determinant of ACLF severity and prognosis, often associated with systemic inflammation. The lung was chosen due to its involvement in respiratory failure and increased susceptibility to infection in patients with ACLF. Finally, the heart was analyzed because cardiac dysfunction and circulatory collapse are common in advanced ACLF, driven by systemic inflammatory responses.^5^ Including these organs allowed us to capture the systemic nature of ACLF, evaluate immune cell infiltration patterns, and correlate immunopathology with organ-specific failure markers, providing a comprehensive view of disease progression.

Gross examination revealed a fibrotic and early nodular appearance in livers from the CLD/early cirrhosis group, which was even more pronounced in ACLF animals, whereas normal and ALF groups showed smooth liver surfaces (Figure 2C). The liver weight-to-body weight ratio was significantly elevated in the CLD, ACLF, and ALF groups compared with controls, indicating hepatomegaly associated with chronic injury and acute insult (Figure 2D).

Serum biochemical analysis confirmed these observations: alanine aminotransferase, aspartate aminotransferase, and bilirubin levels were markedly increased in CLD animals, reflecting chronic hepatocellular damage, and were further exacerbated in ACLF, consistent with severe hepatic decompensation (Figure 2D). As expected, ALF mice exhibited a robust elevation of liver injury markers, confirming acute hepatotoxicity (Figure 2D). The progressive increase in liver injury from CLD and ACLF underscores the additive effect of chronic and acute insults in ACLF, supporting its use as a clinically relevant model for mechanistic and therapeutic studies.

**Figure 3:**
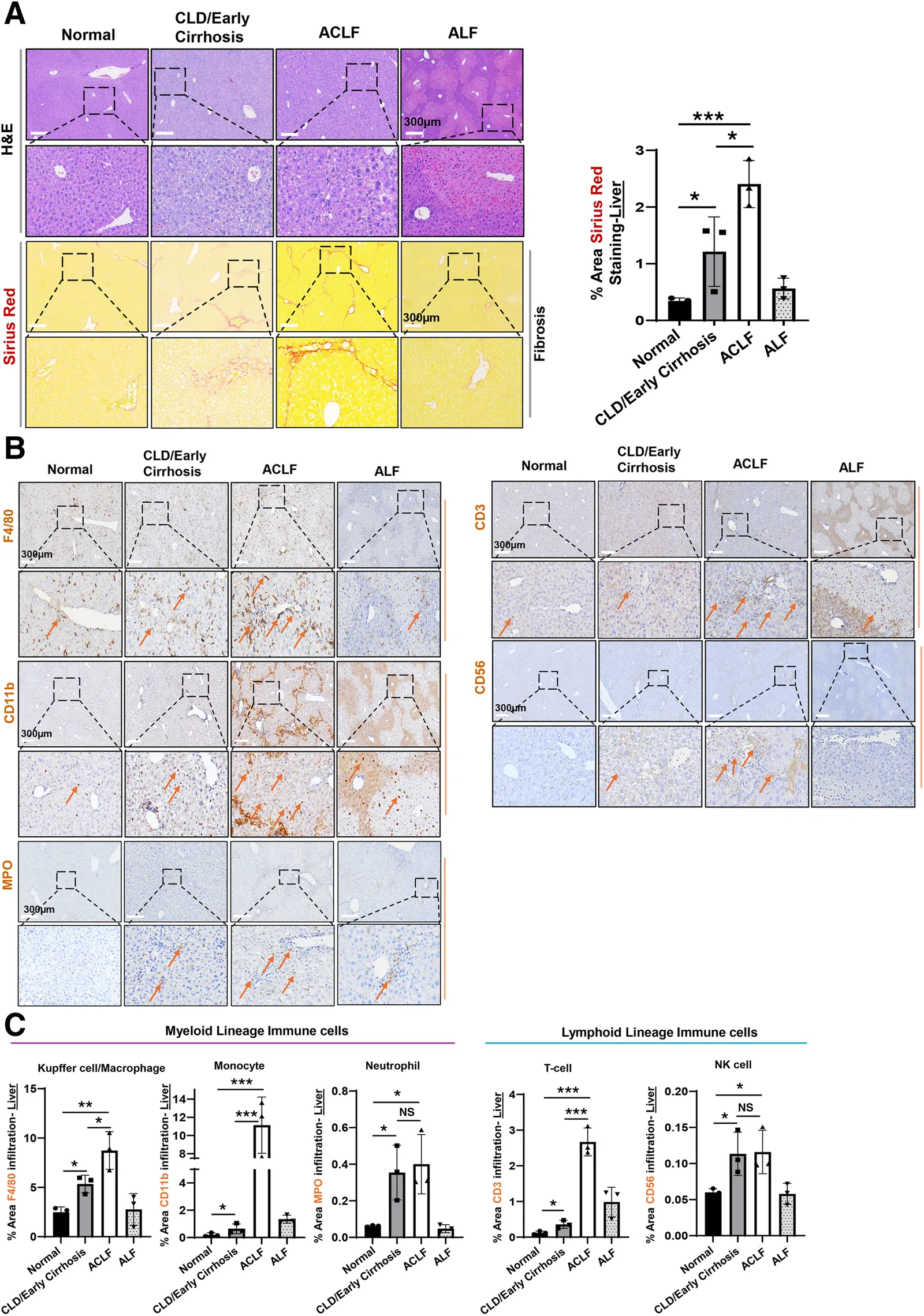
Exacerbated liver injury–associated pathologies in acute-on-chronic liver failure (ACLF). **A:** Representative hematoxylin and eosin (H&E) and Sirius Red staining of liver sections; bar graph shows quantification of Sirius Red–positive area (fibrosis extent). **B:** Myeloid immune cell infiltration assessed by immunohistochemistry for F4/80, CD11b, and myeloperoxidase (MPO), with corresponding quantification. **Arrows** indicate regions of positive staining for myeloid (F4/80, CD11b, and MPO) and lymphoid (CD3 and CD56) immune cells. **C:** Lymphoid lineage immune cell infiltration assessed by CD3 (T cells) and CD56 (natural killer [NK] cells) immunohistochemistry and quantification. Data are expressed as means ± SEM. *n* ≥ 3 per group. ∗*P* < 0.05, ∗∗*P* < 0.01, ∗∗∗*P* < 0.001. Scale bars = 300 μm (**A** and **B**). Original magnification, ×16 (**A** and **B**, **enlarged boxed areas**). ALF, acute liver failure; NS, not significant.

Histologic analysis (hematoxylin and eosin staining) revealed distinct patterns of liver injury across groups (Figure 3A). ACLF exhibited the most severe architectural disruption, characterized by extensive hepatocellular necrosis and large confluent areas of cell death, indicating a progressive decline in liver function compared with CLD. The CLD/early cirrhosis group showed only mild to moderate architectural distortion, with hepatocyte ballooning degeneration and occasional inflammatory foci, while necrosis remained limited. In contrast, ALF livers displayed diffuse hepatocyte necrosis and apoptosis, accompanied by prominent parenchymal collapse and loss of hepatic cords. Infiltration of non-parenchymal cells was evident in CLD and markedly increased in ACLF. ALF livers exhibited extensive necrotic regions around the central vein, indicating massive hepatocyte death, a feature absent in ACLF. These findings underscore that ACLF and ALF are histologically distinct and that ACLF represents a more severe stage of CLD, reflecting progressive deterioration of liver architecture and function.

Fibrosis assessment using Sirius Red staining displayed significantly greater fibrotic deposition in ACLF compared with CLD/early cirrhosis and all other groups (Figure 3A). Quantitative analysis of Sirius Red–positive areas confirmed this difference, with ACLF livers displaying the highest percentage of fibrotic tissue (*P* < 0.05 vs CLD/early cirrhosis; *P* < 0.0001 vs ALF and controls). Fibrotic areas were absent in ALF and control livers. These findings indicate that ACLF represents a disease stage in which chronic fibrosis is compounded by acute hepatic injury, resulting in a dual-hit pathology. This contrasts with ALF, which lacks fibrosis because it develops on a previously healthy liver. The presence of extensive fibrosis in ACLF animals further validates this model as clinically representative.^24^

To determine whether liver injury was associated with immune cell recruitment, hepatic infiltration of both myeloid and lymphoid populations was analyzed. Although the liver normally contains resident Kupffer cells, ACLF livers exhibited prominent infiltration of monocytes and neutrophils (myeloid lineage) (Figures 3, B-C). Lymphoid cells, including CD3-positive T lymphocytes and CD56-positive natural killer cells, were also increased in ACLF (Figures 3, B-C). Inflammatory infiltration was observed in ALF but was less extensive than in ACLF and largely restricted to necrotic zones. Quantitative analysis confirmed that myeloid cell infiltration was significantly higher than lymphoid cell infiltration, suggesting that myeloid cells are the predominant pathologic drivers of ACLF progression (Figures 3, B-C). This predominance of myeloid infiltration further indicates an imbalance between innate and adaptive immunity.

Collectively, these findings validate the establishment of distinct disease states (CLD/early cirrhosis, ACLF, and ALF) and show that ACLF represents the most severe phenotype, characterized by hepatic inflammation, chronic fibrosis, and superimposed acute necrosis.

### Extrahepatic Histologic Signature of Multiorgan Dysfunction and Failure in ACLF

**Figure 4:**
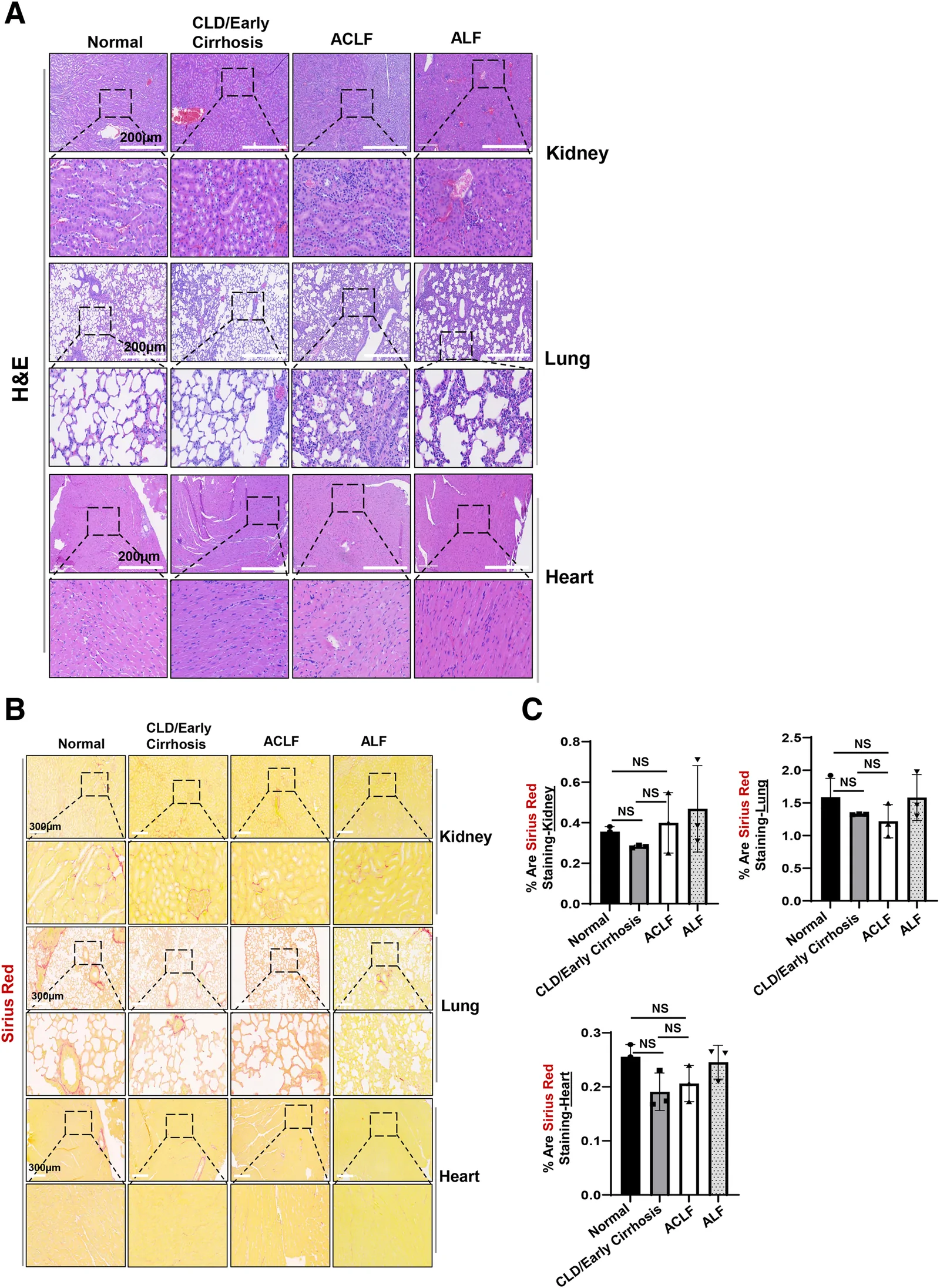
Extrahepatic histopathologic features of multi-organ dysfunction in acute-on-chronic liver failure (ACLF). **A:** Representative hematoxylin and eosin (H&E)-stained sections of kidney, heart, and lung from the normal, chronic liver disease (CLD)/early cirrhosis, ACLF, and acute liver failure (ALF) groups. **B** and **C:** Sirius Red staining of kidney, heart, and lung sections indicates minimal fibrosis across groups, with only modest collagen deposition near vascular niches. Bar graph plot show the quantification of Sirius Red–positive area shows fibrosis extent in kidney, heart, and lung. Data are expressed as means ± SEM. *n* ≥ 5 per group. Scale bars: 200 μm (**A**); 300 μm (**B**). Original magnification: ×8 (**A**, **enlarged boxed areas**); ×16 (**B**, **enlarged boxed areas**). NS, not significant.

To determine whether the CALPS model exhibits extrahepatic features of multi-organ dysfunction, histologic analysis of kidney, lung, and heart tissues was performed. Hematoxylin and eosin staining revealed distinct pathologic changes in ACLF compared with other groups (Figure 4A).

ACLF kidneys displayed severe alterations, including widespread tubular epithelial cell necrosis, loss of brush border integrity, glomerular congestion, and capillary dilation, accompanied by marked interstitial edema (Figure 4A). In contrast, CLD/early cirrhosis kidneys appeared largely normal, with preserved glomerular and tubular architecture, displaying only occasional mild vascular congestion. ALF kidneys exhibited mild to moderate tubular degeneration and focal necrosis, but these changes were less extensive than in ACLF, and the glomerular structure remained relatively intact (Figure 4A).

ACLF lungs exhibited severe pathologic changes characterized by thickened alveolar septa, marked interstitial edema, vascular congestion, microhemorrhages, and collapsed alveolar spaces, indicating impaired gas exchange and systemic inflammatory injury (Figure 4A). CLD lungs showed only mild vascular congestion and minimal interstitial changes. ALF lungs exhibited severe interstitial edema and extensive inflammatory infiltration (Figure 4A), sometimes even more pronounced than in ACLF, highlighting acute systemic involvement in ALF.

ACLF hearts displayed noticeable pathologic alterations, including disruption of myocardial fiber alignment, and signs of vascular congestion (Figure 4A). These features are consistent with cardiac changes that can occur in the setting of systemic inflammation and circulatory stress. Conversely, myocardial fibers in CLD and ALF groups remained largely intact, with minimal structural changes and negligible immune infiltration (Figure 4A). This suggests that significant cardiac involvement is unique to ACLF.

Sirius Red staining across the kidney, lung, and heart revealed no remarkable differences among groups, except for modest collagen deposition around vascular niches (Figures 4, B-C). These findings indicate that extrahepatic organ failure in ACLF is primarily driven by acute inflammatory and immune-mediated injury rather than fibrotic remodeling, reinforcing the need for immunomodulatory interventions rather than antifibrotic strategies.

### CALPS Model of ACLF Has Systemic Inflammation and Signature of Myeloid Lineage Immunopathology

Immune cell infiltration into tissues drives the immunopathology that contributes to MOF and poor outcomes in ACLF. However, whether preclinical ACLF models recapitulate the immune signatures observed in patients remains unclear. To address this, whether the CALPS model reflects the complex interplay of innate and adaptive immune dysfunction seen clinically was examined, thereby validating its utility for mechanistic studies and therapeutic testing.

F4/80–positive macrophages in kidney, lung, and heart tissues were first assessed. F4/80 is a well-characterized cell surface glycoprotein and a canonical marker for murine macrophages and certain tissue-resident myeloid cells. It belongs to the EGF-TM7 family of adhesion G protein–coupled receptors and is predominantly expressed on mature macrophages, including Kupffer cells in the liver and macrophages in other tissues such as the kidney, lung, and heart.^25^ Functionally, F4/80 plays a role in immune regulation, particularly in peripheral tolerance and macrophage-mediated immune responses.

**Figure 5:**
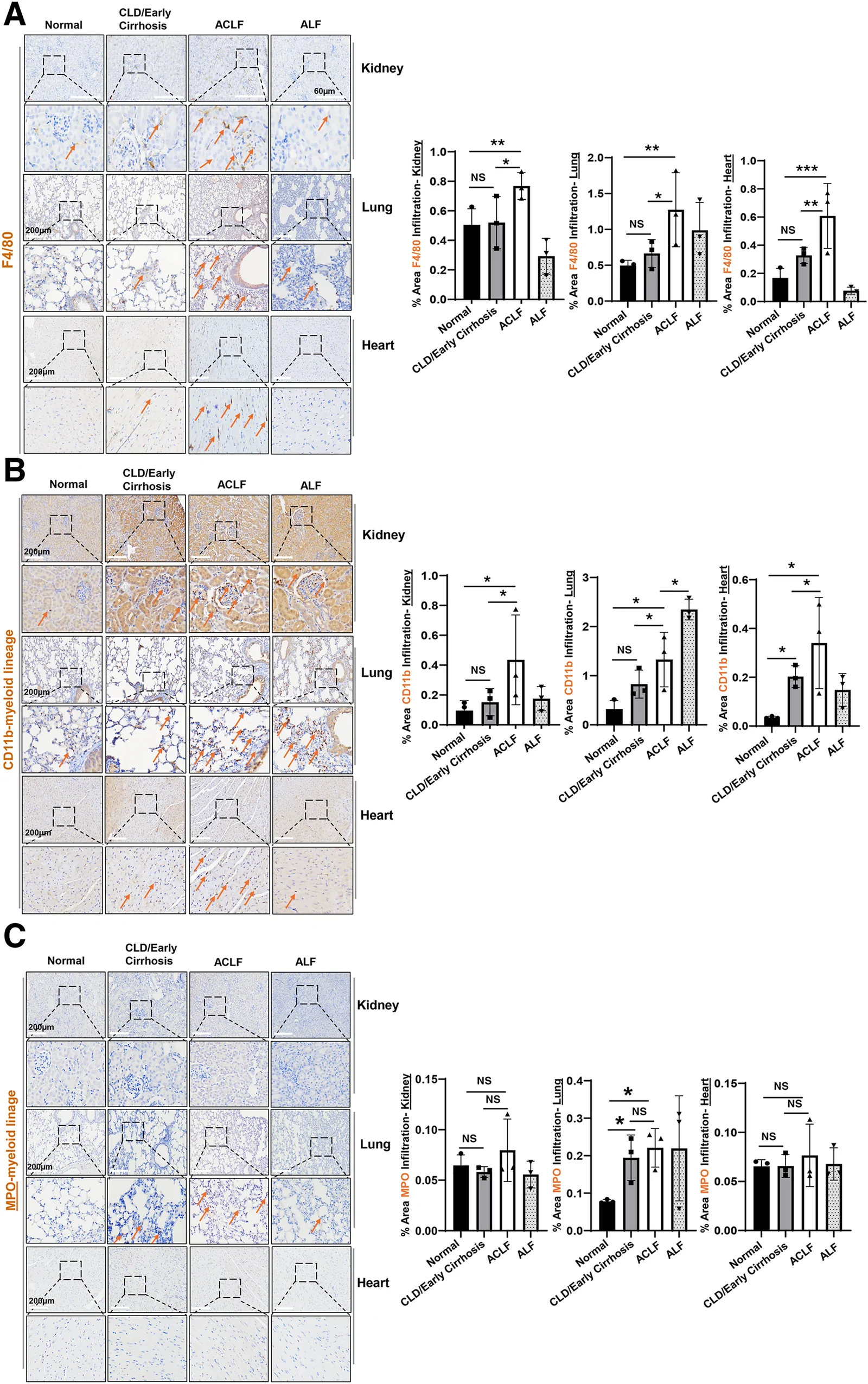
Systemic inflammation and myeloid cell pathology in the CALPS model of acute-on-chronic liver failure (ACLF). **A:** Immunohistochemistry for F4/80–positive macrophages in kidney, lung, and heart across experimental groups [normal, chronic liver disease (CLD)/early cirrhosis, ACLF, and acute liver failure (ALF)]. ACLF tissues exhibit marked macrophage infiltration compared with other groups. Bar graphs show the percentage of F4/80–positive area in each tissue. **Arrows** indicate regions of positive immunostaining for F4/80–positive macrophages in the histologic images. **B:** CD11b staining reveals robust recruitment of inflammatory myeloid cells. ACLF kidneys display dense CD11b-positive infiltrates in glomeruli and interstitium, with moderate staining in the CLD/early cirrhosis and ALF groups. Heart tissue shows selective CD11b-positive infiltration in ACLF; the lungs display progressive infiltration from CLD to ACLF, peaking in ALF. Quantification is shown as percentage of CD11b-positive area. **Arrows** indicate regions of positive immunostaining for CD11b-positive myeloid immune cells in the histologic images. **C:** Myeloperoxidase (MPO) staining indicates increased neutrophil infiltration in ACLF kidneys and lungs, with minimal presence in heart and other groups. Bar graphs represent MPO-positive area percentages. **Arrows** indicate regions of positive immunostaining for MPO-positive neutrophils in the histologic images. Data are expressed as means ± SEM. *n* ≥ 5 per group. ∗*P* < 0.05, ∗∗*P* < 0.01, ∗∗∗*P* < 0.001. Scale bars: 60 μm (**A**, **top panels**), 200 μm (**A**, **other panels**); 200 μm (**B** and **C**). Original magnification: ×6 [**A** (**kidney**), **enlarged boxed areas**]; ×8 [**A** (**lung and heart**), **B**, and **C**, **enlarged boxed areas**]. NS, not significant.

Immunohistologic analysis revealed marked F4/80 staining in all three organs of ACLF mice, whereas staining was comparable between the CLD/early cirrhosis and ALF groups (Figure 5A). These findings suggest an ACLF-specific elevation of F4/80–positive macrophages in extrahepatic tissues, supporting the presence of immunopathology in this model.

Next, CD11b, a marker of inflammatory myeloid cells such as monocytes, macrophages, and neutrophils, was examined.^26^ CD11b is also known as integrin αM (part of the Mac-1 complex), and increased CD11b staining indicates recruitment and accumulation of inflammatory myeloid cells in tissues. CD11b staining was markedly increased in ACLF kidneys, with infiltrates observed in both glomerular units and interstitial regions, indicating intense myeloid-driven inflammation (Figure 5B). In contrast, CLD and ALF kidneys showed only mild to moderate CD11b-positive cells, primarily localized to glomeruli. In heart tissue, CD11b-positive cells were significantly elevated only in ACLF, with minimal staining in CLD and ALF, suggesting that cardiac involvement is a unique feature of ACLF immunopathology (Figure 5B). Lung tissue analysis revealed increased CD11b-positive cells in CLD compared with controls, with even greater infiltration in ACLF lungs. Interestingly, ALF lungs exhibited the highest CD11b infiltration, consistent with acute systemic inflammation in this group. These findings suggest that although ALF lungs experience acute immune infiltration, ACLF lungs sustain a chronic and extensive inflammatory burden, reinforcing the systemic nature of ACLF immunopathology.

To further characterize myeloid-driven pathology, neutrophil infiltration was assessed using MPO staining. Neutrophils are key mediators of acute inflammation and contribute to tissue injury through the release of reactive oxygen species, proteolytic enzymes, and pro-inflammatory cytokines.^27^ Their presence in extrahepatic organs during ACLF would indicate an aggressive inflammatory response extending beyond the liver. Immunohistologic staining for the neutrophil marker MPO revealed either absent or modest staining in normal, CLD/early cirrhosis, and ALF tissues. In contrast, ACLF kidneys and lungs exhibited increased MPO staining, whereas the heart showed no significant infiltration (Figure 5C). Although MPO intensity was lower than that of F4/80 or CD11b, these findings indicate that neutrophils contribute to systemic inflammation in ACLF, along with macrophages and monocytes as key drivers of tissue immunopathology.

Collectively, these results show that the CALPS model of ACLF exhibits systemic inflammation characterized by widespread myeloid lineage infiltration, validating its relevance for studying immunopathology and therapeutic interventions.

### CALPS Model of ACLF Exhibits Lymphoid Lineage Immunopathology in Extrahepatic Organs

In addition to myeloid-driven inflammation, ACLF is associated with dysregulation of lymphoid immune cells, which contributes to systemic immunopathology and MOF.^28^ Lymphoid cells, including T cells (CD3) and natural killer cells (CD56), are key components of adaptive immunity, and their infiltration into extrahepatic organs reflects a breakdown of immune compartmentalization and systemic immune activation.

**Figure 6:**
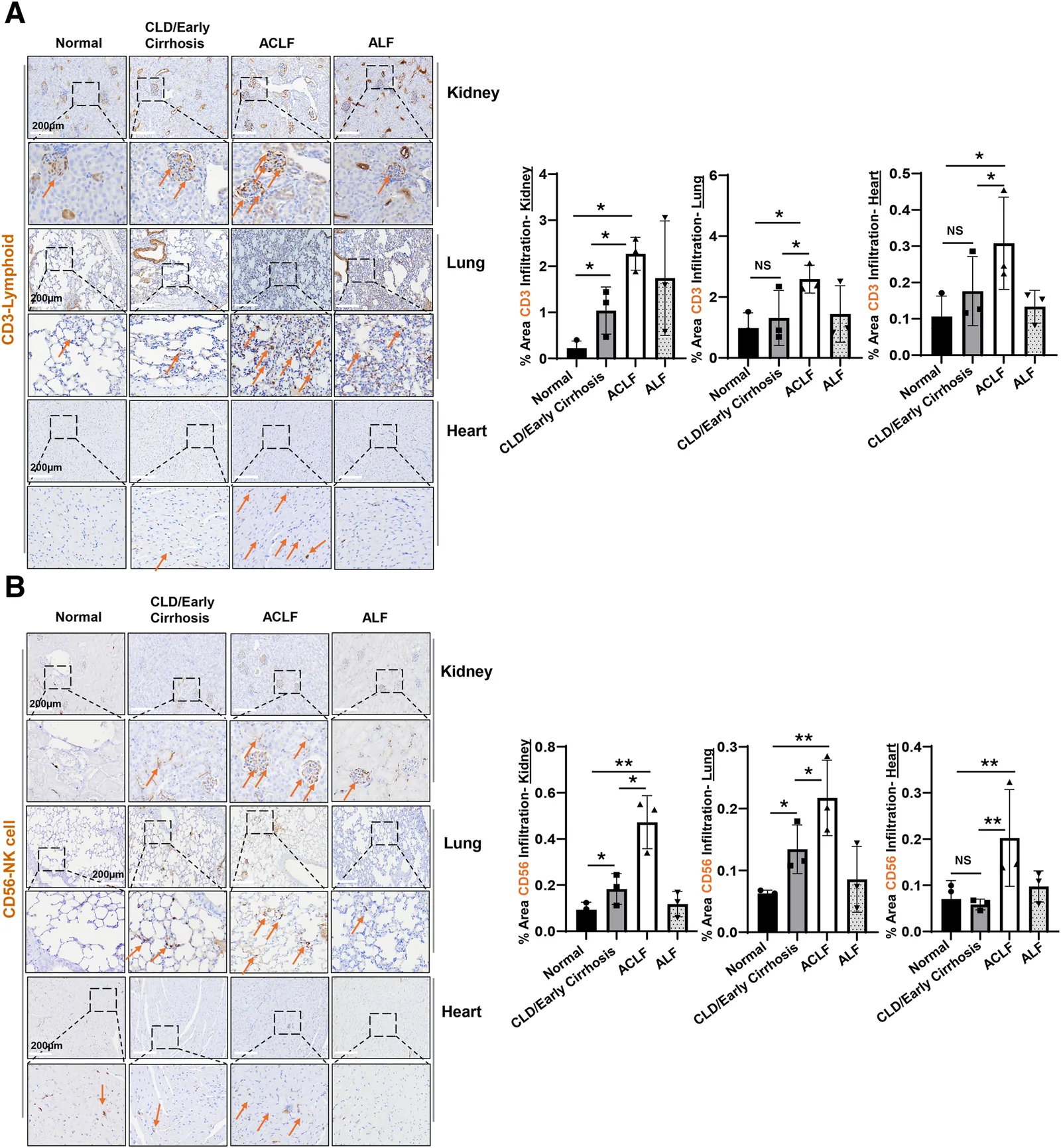
CALPS model of acute-on-chronic liver failure (ACLF) exhibits lymphoid lineage immunopathology in extrahepatic organs. **A:** Immunohistochemical staining for CD3-positive T cells in kidney, lung, and heart sections from normal, chronic liver disease (CLD)/early cirrhosis, ACLF, and acute liver failure (ALF) groups. ACLF tissues show marked infiltration in kidney and lung interstitial regions, with sparse but detectable CD3-positive cells in the heart. Bar graph shows the quantification of CD3-positive area in extrahepatic tissues across experimental groups. **Arrows** indicate the CD3-positive cells. **B:** Immunohistochemical staining for CD56-positive natural killer (NK) cells in liver, kidney, lung, and heart sections from the same groups. NK cell infiltration is modest in lung tissue of the CLD/early cirrhosis and ACLF liver but largely absent in other extrahepatic organs. Bar graph shows the quantification of CD56-positive area in liver and extrahepatic tissues across experimental groups. **Arrows** indicate the CD56-positive cells. Bar graphs represent means ± SEM. *n* ≥ 5 per group. ∗*P* < 0.05, ∗∗*P* < 0.01. Scale bars = 200 μm (**A** and **B**). Original magnification, ×8 (**A** and **B**, **enlarged boxed areas**). NS, not significant.

To assess this, immunohistologic staining was performed for lymphoid markers in kidney, lung, and heart tissues from ACLF, ALF, CLD/early cirrhosis, and normal control groups. ACLF tissues exhibited significant infiltration of CD3-positive T cells in kidney and lung interstitial regions (Figure 6A). The heart showed sparse but detectable CD3-positive cells only in ACLF, suggesting selective cardiac involvement (Figure 6A). CD56-positive natural killer cells were modestly elevated in lung tissue of CLD/early cirrhosis and in ACLF liver but were largely absent in other extrahepatic tissues (Figure 6B), suggesting a minor role in systemic immunopathology.

These findings show that T cell–mediated immunopathology dominates systemic inflammation in ACLF, whereas natural killer cell involvement remains limited. The coexistence of lymphoid infiltration with myeloid activation underscores the complexity of ACLF immunopathology and validates the CALPS model as a clinically relevant platform for studying immune-driven MOF.

### Myeloid and Lymphoid Lineage Immunopathology Correlates with Kidney Dysfunction and Failure in ACLF

The kidney consistently exhibited extensive infiltration of both myeloid (F4/80 positive and CD11b positive) and lymphoid (CD3 positive) immune cells in the CALPS model of ACLF (Figures 5 and 6). Given that renal failure is the most frequent organ dysfunction in patients with ACLF^29^ and that the kidney plays a pivotal role in systemic homeostasis, such immune cell accumulation may compromise renal function and exacerbate MOF. Therefore, the kidney was selected as a representative organ for detailed analysis of multi-organ dysfunction.

**Figure 7:**
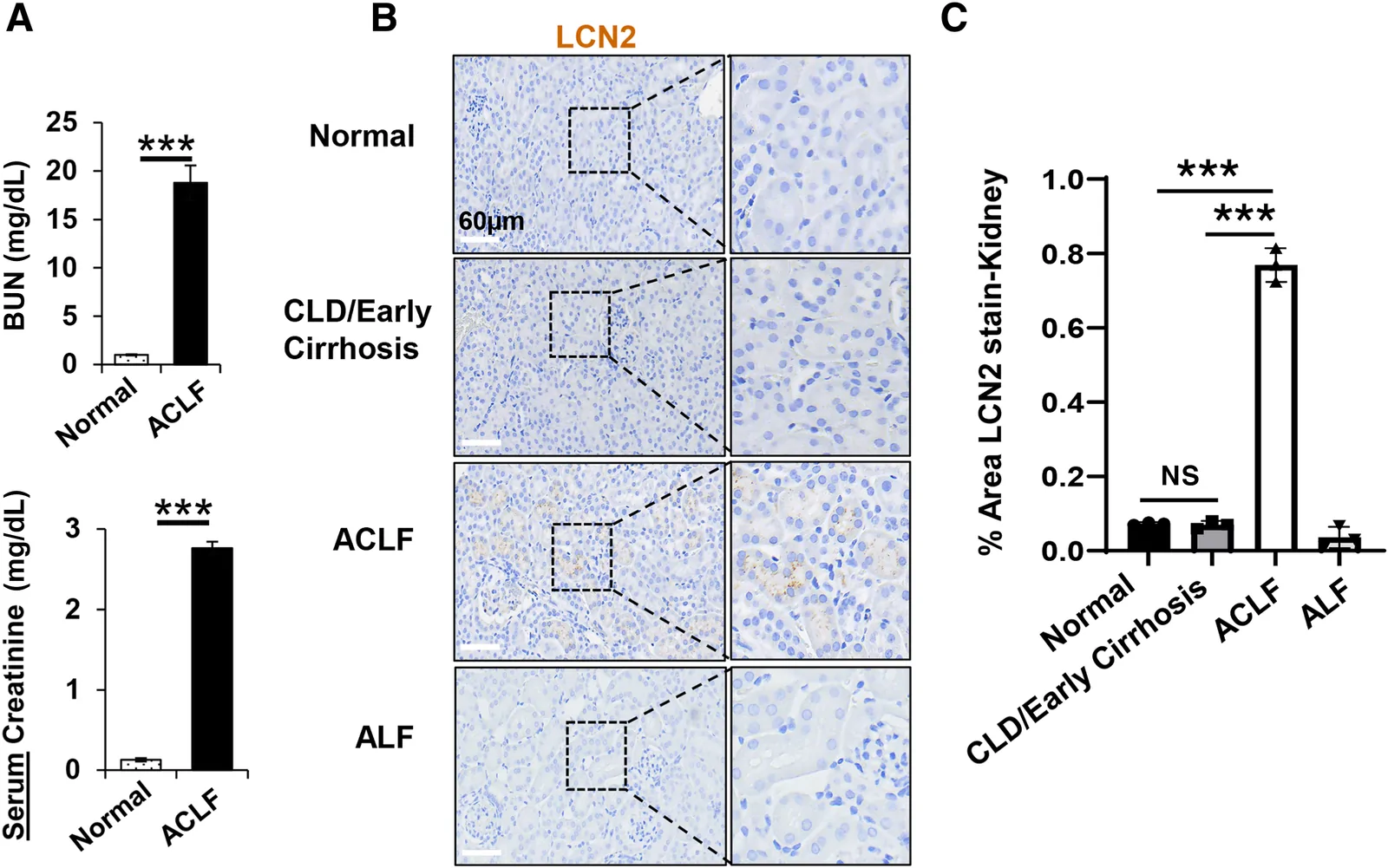
Immune infiltration in kidney correlates with renal dysfunction in acute-on-chronic liver failure (ACLF). **A:** Blood urea nitrogen (BUN) levels and serum creatinine across experimental groups, indicating impaired renal function in ACLF mice. **B:** Immunohistochemical staining for lipocalin-2 (LCN2), a marker of acute kidney injury, in kidney sections from normal, chronic liver disease (CLD)/early cirrhosis, ACLF, and acute liver failure (ALF) groups. ACLF kidneys exhibit strong LCN2 staining in tubular epithelial cells, whereas other groups show minimal or no staining. **C:** Quantification of LCN2-positive area in kidney sections across groups. Data are expressed as means ± SEM. *n* ≥ 5 per group. ∗∗∗*P* < 0.001. Scale bars = 60 μm (**B**). Original magnification, ×6 (**B**, **enlarged boxed areas**). NS, not significant.

Biochemical assessment revealed a significant elevation of blood urea nitrogen and serum creatinine levels in ACLF mice compared with the normal, CLD/early cirrhosis, and ALF groups, indicating impaired renal function (Figure 7A). Histopathologic examination confirmed structural damage, including tubular degeneration and glomerular alterations, in ACLF kidneys (Figure 4A). To further validate renal injury, immunohistologic staining was performed for LCN2, a sensitive and widely used biomarker of acute kidney injury that reflects tubular epithelial stress and damage.^30^^,^^31^ ACLF kidneys exhibited strong LCN2 staining in tubular epithelial cells, whereas the normal, CLD/early cirrhosis, and ALF groups showed minimal or no staining (Figures 7, B and C).

These findings highlight the pathogenic role of systemic immunopathology in ACLF, showing that immune-driven injury extends beyond the liver and contributes to MOF. Targeting immune-mediated pathways may therefore represent a promising therapeutic strategy to prevent or mitigate organ dysfunction in ACLF.

## Discussion

**Figure 8:**
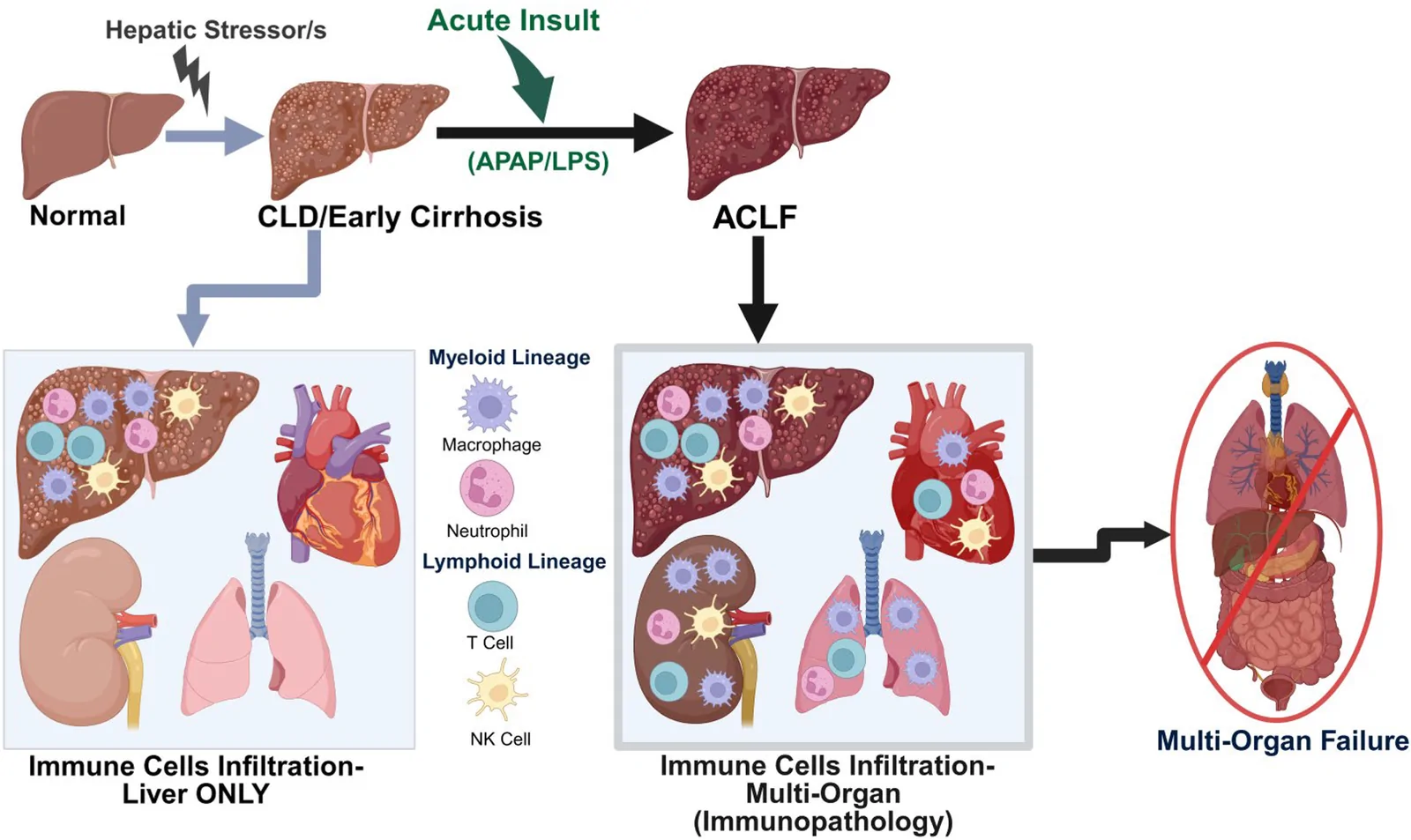
Schematic representation of immunopathology and multi-organ dysfunction in the CALPS model of acute-on-chronic liver failure (ACLF). Normal liver exposed to chronic hepatic stressors progresses to chronic liver disease (CLD)/early cirrhosis, characterized by localized hepatic immune cell infiltration and hepatitis. An acute insult in the context of CLD/early cirrhosis triggers extensive infiltration of myeloid (F4/80–positive macrophages, CD11b-positive monocytes/neutrophils, and myeloperoxidase-positive neutrophils) and lymphoid (CD3-positive T cells and CD56-positive natural killer cells) immune cells not only in the liver but also in extrahepatic organs, including the kidney, lung, and heart, resulting in systemic immunopathology. This widespread immune-driven injury contributes to multi-organ dysfunction and failure in ACLF. Created with BioRender.com (Toronto, ON, Canada).

ACLF is a complex clinical syndrome characterized by systemic inflammation, immune dysregulation, and MOF. Survival in clinical ACLF is closely linked to the number and type of extrahepatic organ failures.^32^ However, the mechanisms driving MOF remain poorly understood, with immunopathology considered a critical contributor. Using the CALPS model, the current study provides mechanistic insights into how immune-driven pathology extends beyond the liver, involving both myeloid and lymphoid lineages, and contributes to extrahepatic organ dysfunction (Figure 8).

The CALPS model was selected for two key reasons: i) it recapitulates hallmark pathologic features of clinical ACLF, including portal hypertension, ascites, and severe hepatic decompensation^15^; and ii) its two-hit design enables comparative analysis across distinct disease states (CLD, ACLF, and ALF) and healthy controls, allowing systematic evaluation of disease progression and immune dysregulation. This flexibility makes the model highly suitable for mechanistic studies and therapeutic testing.

### Immune Infiltration as a Driver of Systemic Immunopathology

Our findings reveal massive infiltration of myeloid cells (F4/80–positive macrophages, CD11b-positive monocytes, and MPO-positive neutrophils) and lymphoid cells (CD3-positive T cells) in extrahepatic organs, particularly the kidney, lung, and heart, in ACLF mice. This dual immune signature mirrors clinical observations in patients with ACLF, in whom hyperinflammation and immune cell redistribution correlate with poor outcomes.^28^ Myeloid cells release pro-inflammatory cytokines, reactive oxygen species, and proteolytic enzymes, whereas T cells amplify inflammation through cytokine signaling and recruitment of additional immune cells.^33^ The interplay between these lineages likely creates a self-perpetuating inflammatory loop, accelerating tissue injury and organ dysfunction.

### Interplay of Myeloid and Lymphoid Immunopathology in ACLF

ACLF is characterized by a dual immune signature involving both innate and adaptive immunity (Figures 5 and 6). Myeloid cells, particularly macrophages and monocytes, showed extensive infiltration into extrahepatic organs, suggesting their dominant role in driving systemic inflammation. Concurrently, lymphoid cells, especially CD3-positive T cells, were markedly elevated (Figure 6A), indicating adaptive immune activation alongside innate immune dysregulation. Reciprocal signaling between these lineages (myeloid-derived cytokines activating T cells and T-cell–mediated recruitment and polarization of macrophages) likely amplifies immunopathology. This synergistic dysfunction may underlie the progression from localized hepatic injury to MOF, a hallmark of ACLF. Understanding this crosstalk is critical for developing therapies that target both immune compartments.

### Link Between Immune Infiltration and Kidney Injury in ACLF

The kidney emerged as a major target of systemic immunopathology in ACLF, exhibiting the highest degree of immune infiltration among extrahepatic organs (Figures 5 and 6). Functional assays exhibited significantly elevated serum creatinine and blood urea nitrogen levels in ACLF mice (Figure 7A), confirming impairment renal function. Histologic analysis further revealed tubular degeneration and glomerular alterations, indicative of structural damage. Complementing these findings, LCN2 staining, an established biomarker of tubular stress and acute kidney injury,^30^^,^^31^ showed robust expression in ACLF kidneys, while remaining minimal or absent in early CLD and ALF models (Figures 7, B and C). These observations suggest that renal dysfunction in ACLF is driven predominantly by immune-mediated injury rather than by hemodynamic perturbations alone.

Infiltrating macrophages and T cells likely drive tubular injury by releasing proinflammatory cytokines, chemokines, and reactive oxygen species, leading to oxidative stress and apoptosis in renal epithelial cells. Circulating mediators, including IL-6, tumor necrosis factor-α, and damage-associated molecular patterns from damaged hepatocytes, may further potentiate this process by sensitizing renal tissue to immune cell–derived cytotoxic signals and disrupting epithelial and endothelial integrity. Together, the combined effects of immune infiltration and systemic inflammatory mediators create a feed-forward inflammatory cycle that underlies the severe kidney injury observed in ACLF.

### Progression from Localized to Systemic Immunopathology

Early-stage CLD models (CCl_4_-induced and Atg7 deletion) displayed immune infiltration confined to the liver, with no detectable involvement of extrahepatic organs (Figure 1). In contrast, ACLF exhibited a striking shift toward systemic immune activation, characterized by widespread infiltration in the kidney, lung, and heart (Figures 5 and 6). This transition from localized hepatic inflammation to multi-organ immunopathology underscores a critical inflection point in disease progression and highlights the need for early therapeutic intervention before systemic immune activation becomes established.

Because the current study focused on a 24-hour post-insult time point, the findings reflect the early phase of systemic immunopathology. At later stages, immune infiltration, cytokine signaling, and tissue injury are likely to intensify or evolve, potentially generating sustained inflammatory circuits that accelerate MOF and mortality in ACLF.

### Clinical and Therapeutic Implications

These study data suggest that ACLF pathogenesis involves coordinated activation of innate and adaptive immune responses, leading to MOF. Therapeutic strategies should therefore aim to modulate both myeloid and lymphoid pathways. Targeting macrophage activation, T-cell recruitment, and cytokine signaling may help prevent immune-driven organ injury. In addition, monitoring biomarkers such as LCN2 could provide early detection of renal involvement in patients with ACLF, enabling timely intervention.

### Limitations

This study had several limitations. First, although a strong correlation between immune cell infiltration and kidney dysfunction was observed, mechanistic experiments were not performed to establish a direct cause-and-effect relationship. Future studies using interventional approaches, such as immune cell depletion or cytokine blockade, will be essential to confirm whether immune infiltration drives organ failure. Second, due to resource constraints, the functional analysis focused primarily on the kidney; functional impairment in other extrahepatic organs such as the lung and heart, which also exhibited immune infiltration, were not comprehensively assessed. Third, sampling was limited to 24 hours’ post-acute insult in ACLF. Evaluating tissues at later time points (eg, day 7, day 11, or beyond) could provide a more complete picture of the temporal dynamics of immune pathology and organ injury in ACLF. Addressing these limitations will strengthen the mechanistic understanding of systemic immunopathology in ACLF.

## Conclusions

The CALPS model faithfully recapitulates the dual immune signature (myeloid and lymphoid infiltration) observed in clinical ACLF and shows its pathogenic role in extrahepatic organ dysfunction. These findings validate the model for mechanistic studies and therapeutic testing, paving the way for immune-targeted interventions to improve outcomes in ACLF.

## Disclosure Statement

None declared.
